# Reaching a Better Understanding of the Control of Bimanual Movements in Older Adults

**DOI:** 10.1371/journal.pone.0047222

**Published:** 2012-10-15

**Authors:** Rachel O. Coats, John P. Wann

**Affiliations:** 1 Centre for Sport and Exercise Sciences, University of Leeds, United Kingdom; 2 Department of Psychology, Royal Holloway University London, United Kingdom; Ecole Normale Supérieure, France

## Abstract

The ability to interact skilfully with the environment is essential for independent living and therefore a critical factor for the aging population. Here we investigate the differences between young and older adults in a bimanual reaching task where the goal is to bring two objects together to the same location with a synchronous placement. Older (mean age 74) and young (mean age 20) adults were asked to pick up two spatially disparate objects, one in each hand, and bring them together to place them in one of three trays laid out in front of them from left to right. The results showed that the older adults were no more detrimentally affected than the young by asymmetric bimanual movements compared to symmetric ones, and both groups completed their movements in the same time. Nevertheless, compared to the young, the older adult group produced reaches characterised by higher peak velocities (although this effect was marginal), shorter hover times, and where the movement distance varied for each hand the scaling of the kinematic profile across the two limbs diverged from that found with younger participants. They then spent longer than the young in the final adjustment phase and during this phase they made more adjustments than the young, and as a result were more synchronous in terms of the final placement of the objects. It seems that the older adults produced reach movements that were designed to reach the vicinity of the tray quite rapidly, after which time they made discreet adjustments to their initial trajectories in order to exercise the precision necessary to place the objects in the tray. These findings are consistent with the idea that older adults have problems using online control (as they wait until they can fixate both objects before making adjustments).

## Introduction

Numerous motor tasks we perform in everyday life rely on moving both hands in a coordinated fashion and performing a bimanual reach. It is commonly observed that adult humans are skilled at coordinating the left and right hands when reaching to grasp two separate objects at the same time [1]. However, bimanual actions include not only those where a movement is made towards an object with the intention of picking it up, but also those in which the person is already holding two objects and moves them simultaneously (often in order to bring them together). It is not yet clear how older adults coordinate their movements in such bimanual tasks, especially those requiring high degrees of precision. This paper addresses this issue.

Historically there has been some debate over whether or not the two arm movements comprising a bimanual reach are simultaneous. While a number of studies have shown that participants tend to temporally synchronise movements in incongruent bimanual reaching [2] and aiming [3] more recent evidence suggests that this is not the case. Kelso et al. [2] and Jackson et al. [3] postulated that the limb coupling they found provides evidence for the idea that the limbs act as a functional, synergistic unit. In contrast, others found evidence for asynchronous timing during incongruent movements [1], [4], [5], [6], [7] and argued in favour of independent control systems, albeit allowing neural crosstalk [5], [8] causing the movement of one hand to affect the movement of the other.

Explanations have been put forward in an attempt to resolve these contrasting views. Miller and Smyth [6] and Mason and Bruyn [5] pointed out that if movements were examined at the level of each individual trial asynchrony would be evident, but this is often hidden by the examination of movement kinematics averages. Riek et al [4] pointed out that close temporal proximity between the start and end of the two hand movements should not necessarily be taken as evidence for synchronous timing throughout the reach (as it has been by Kelso et al [2]). Reaches might end at the same time but the kinematics of each reach might differ up until this point. For example, they discovered what they termed a ‘hover phase’ at the end of the movement. One hand would be moved to the target and wait whilst corrections were made to the position of other hand before they were simultaneously lowered. Riek et al [4] went on to suggest that synchrony is highly task-dependent. Mason and Bruyn [5] also support the idea of synchrony being task-dependent and suggested that there is a *functional coupling* of the upper limbs such that the hands and arms can be coupled when required, but are also capable of performing independently.

If synchrony is highly task-dependent then what is it about one task that makes it so different from another? Recent evidence has shown it could be a question of visual guidance. The importance of vision in bimanual reaching was evident in Riek et al’s [4] study. The hover phase at the end of the movement reflected the fact that both targets could not be fixated at the same time. One target was fixated and endpoint errors corrected for the hand moving to that target, before fixation was switched to the other target and errors corrected for that hand. Visual monitoring of both hands was required, leading to temporal asynchronies. The authors concluded that eye movements must be considered an important constraint in bimanual aiming tasks. Bingham et al [1] also support the idea that asynchronies in bimanual movements are driven by a need to visually guide each hand in turn because two objects that are sufficiently separated cannot be simultaneously fixated. They found a tendency for the hands to be relatively synchronous during the deceleration phases of the reaches, but if task difficulty was increased (target size reduced or the separation between targets increased), the limbs became increasingly uncoupled. They postulated that this was due to the fact that when task difficulty was increased visual guidance became critical to preserve accuracy, and this lead to reduced temporal coupling between the hands.

Inherent in the suggestions of Bingham et al [1] and Riek et al [4] is the idea that overt shifts in visual attention from one hand to the other are required in bimanual movements. If, as evidence suggests (e.g. [1]), asynchrony in bimanual reaching is, at least in part [6], [7] a function of the need for each hand to be visually guided to its target location, we predict that older adults might produce functionally different reaches (perhaps manifested in terms of greater asynchrony) from their younger counterparts due to an over-reliance on, or inefficient use of, visual feedback to guide reaching. In a unimanual reaching study [9] we found that older adults appeared to rely to a greater extent on online visual control, and hence needed to pay close visual attention to the task, to perform at similar levels to the younger participants. Seidler-Dobrin and Stelmach [10] found that older participants spent longer in the secondary submovement of a unimanual reach, which they suggest reflects inefficient use of visual feedback information. In a bimanual task where the target objects are sufficiently far apart from each other one cannot foveate both hands at the same time until they have come together. In this case older adults might have more difficulties performing the task due to a need to fixate both hands but an inability to do so. They might be forced to rely on feedforward strategies or alternative forms of feedback such as proprioception. This is consistent with findings that interlimb coordination performance in older adults is correlated with additional brain activation in sensorimotor and frontal (cognitive) areas rather than just motor regions [11].

Stelmach, Amrhein and Goggin [12] examined bimanual control in older adults. Ten older and ten young participants performed a unimanual task, a symmetric (equal amplitude) bimanual task, and an asymmetric (unequal amplitude) bimanual task. The young participants completed the movement in significantly less time than the older adults in all conditions, and movement time (MT) increased proportionally more for the older adult group than the young with increases in task complexity (although this was due to the differences between unimanual and bimanual movements rather than between the two different (symmetric vs. asymmetric) bimanual movements). The older adults were half as synchronous in initiating and terminating the two hands in the bimanual movements as the young were. Seidler and Stelmach [13] suggested that this overall increase in asynchrony indicates that older adults lose some of their ability to regulate movements using online feedback whilst carrying out the reach. Moving asynchronously would allow them to look from one object to the other. Interestingly, in Stelmach et al’s [12] study both age groups were similarly more asynchronous in starting and terminating asymmetric compared to symmetric movements.

It is important to point out that the participants in Stelmach et al’s [12] study were not specifically told to finish their bimanual movements at the same time. It is possible that the older adults might have done so if instructed. For this reason we have decided to examine performance when synchronous placement is required and participants are instructed to try and place the objects in the specified tray simultaneously. This instruction goes against methods employed in most previous research on bimanual prehension in which participants are generally not given any instruction on how to move. Older adults may well be capable of moving more synchronously when instructed to do so; something we feel is worthy of investigation.

In the following experiment older and young participants made symmetric (movements across equal distances) and asymmetric (movements across unequal distances) bimanual movements to bring two objects together and place them in a specified location. The paradigm used is similar to that of Stelmach et al [12] but, where they had participants make pointing movements and accuracy was not recorded, here precision is key. Given previous findings [12] we predict that, firstly, the older adults will produce different reaches compared to the young, possibly manifested in terms of longer movement times and increased asynchrony, because they will suffer more by not being able keep both hands under constant visual control. Secondly, the synchrony of the movements produced by the older adults will not be detrimentally affected to a greater extent than that of the young by asymmetric movements.

## Methods

### Ethics Statement

The project was scrutinised, according to procedures specified by the University of Reading Ethics and Research Committee, approved and allowed to proceed. Written informed consent was obtained from all participants.

### Participants

Twenty three participants took part in the study. Twelve were recruited from the School of Psychology’s Aging Panel at the University of Reading and were remunerated £5 for their travel expenses. They were between seventy and eighty in age (mean age = 73.75) and six were female and six were male. One participant was identified as an outlier (outside the data range using the boxplot function in SPSS) in four separate dependent variables and therefore removed from the study completely to err on the side of caution and reduce the likelihood of a Type 1 error in analyses. The other eleven participants were students at the University of Reading who took part in the study on a voluntary basis or for credit as part of their undergraduate degree course (9 female, 2 male, mean age = 20.18). All were self-declared right-handers (for ease of analysis) and had normal or corrected-to-normal eyesight. None had any overt movement problems.

### Apparatus

**Figure 1:**
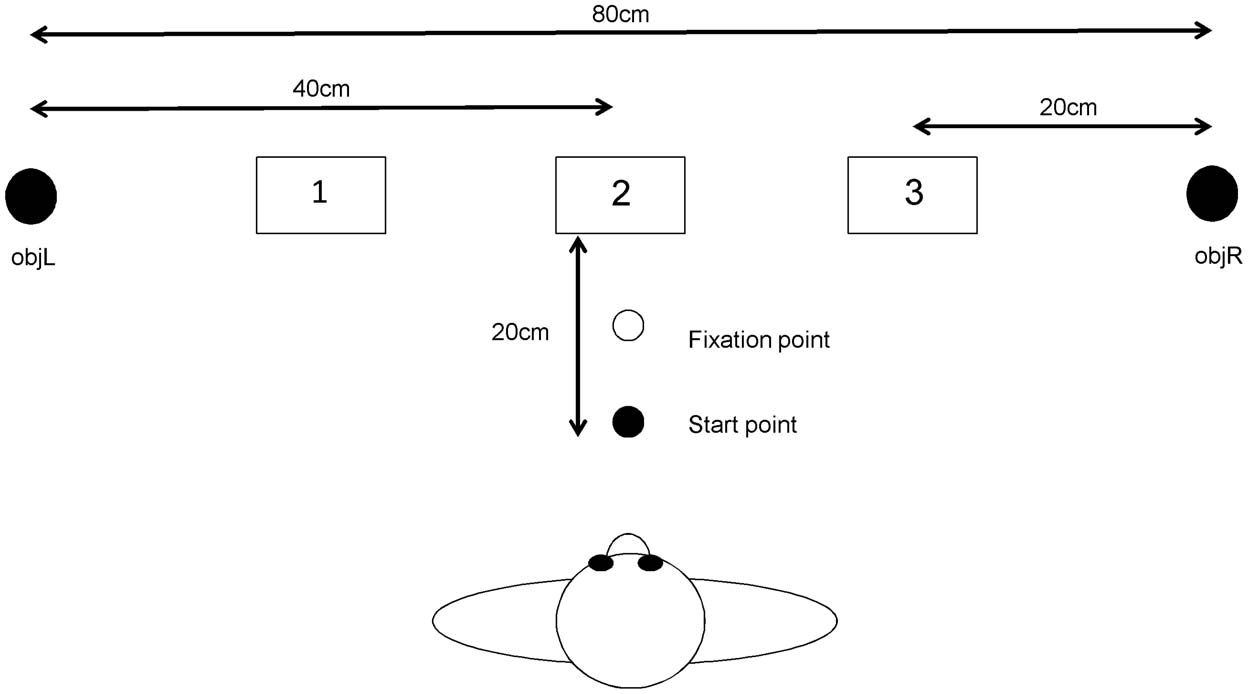
Diagram of experimental setup. Shows the layout of the objects and trays. Participants would start each trial with their hands resting on the start point and looking at the fixation point until one of the trays lit up. The participant would pick up objL in their left hand and objR in their right hand and place them both in the specified tray.

Participants were seated in front of a table, on which were three trays laid from left to right (see Figure 1). All trays were 10×6×2.5 cm in size and were located 20 cm apart from each other. At the start of each trial one of the target objects (objL) was located 20 cm to the left of the left-hand tray (tray 1), and the other target object (objR) was located 20 cm to the right of the right-hand tray (tray 3). This meant that the participant had to move objL 20 cm to tray 1, 40 cm to tray 2 and 60 cm to tray 3. Likewise, objR had to be moved 20 cm to tray 3, 40 cm to tray 2 and 60 cm to tray 1. On any given trial both objects were moved to the same tray. Movements to tray 2 are symmetric as both hands move the same distance to reach the tray, while movements to trays 1 and 3 are asymmetric as the hands move different distances.

The two target objects were identical. They were 14 cm high cylinders that were 4.4 cm in diameter and weighed 200 g. Both objects could be placed into one of the trays at the same time but with only a few millimetres either side. The participant started each trial with both hands on the start point located directly in front of them (and in line with the middle of tray 2), 8 cm from the edge of the table nearest them and 20 cm from the edge of the middle tray. From here all three trays and both objects were within easy reaching distance.

A projector and mirror were used to allow the trays on the table to be lit from underneath. The surface of the table was an opaque sheet and the mirror was angled underneath the surface in such a way that the lights could be projected onto the surface underneath where the objects and trays were located. A LabView programme was written in order to specify which tray the participant should move the objects to by illuminating the appropriate tray for each trial.

The movements of the target objects were recorded by a VICON 3D motion camera system which consists of a data station, six infrared cameras running at 120 Hz, and reflective markers. A marker was placed on the top of each target object to track its position. Data analysis was carried out via a custom written Matlab programme. Calibration of the system was performed at the beginning of each testing session.

### Procedure

Participants started with both hands on the start point in front of them and were told to look at a fixation point located on the table between the start point and tray 2 until the trial started. One of the trays was then lit up and participants were required to pick up the target objects, objL in the left hand and objR in the right hand, and move them both to the specified tray. Only the objects had markers on them (participants’ hands did not) so movements to the objects were not recorded. Participants were told to perform natural reaches and that the key was to try and get the objects to arrive in the tray at the same time. Participants completed 30 trials, 10 to each tray. The order in which the trays were lit up was pseudo-randomised so the participant never moved to the same tray twice in succession.

### Design

**Figure 2:**
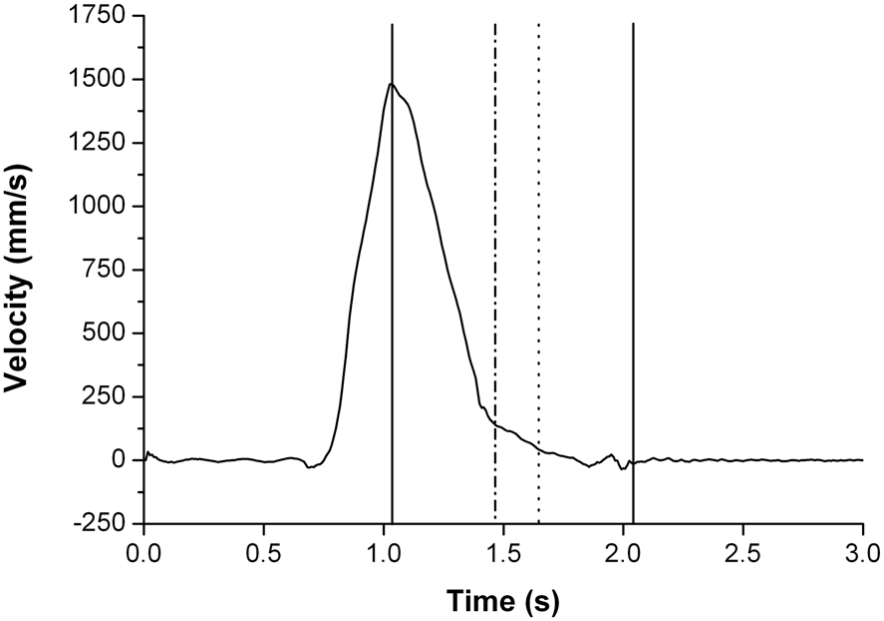
Example velocity profile to show kinematic landmarks. The two vertical solid lines represent the time of peak velocity and the end of the movement. Deceleration time is the time between these two lines. The dot-dash line shows the start of the hover phase (10% of peak velocity). The dotted line represents the end of the hover phase and the start of the final adjustment (FA) phase (3% of peak velocity). It is defined as the finish of the approach to the object. The FA phase ends at the solid line representing the end of the movement.

Along with the traditional kinematic variables we also introduce some new ones. Specifically PVcorr, a measure to give us an estimate of how similar the central motor command structure was for both limbs; hover time, a section close to the end of deceleration time where the participant is moving slowly; and final adjustment time, a section right at the end of deceleration time (following hover time) where the hand has almost stopped and final adjustments are made to the trajectory (see Figure 2). Detailed descriptions are provided below.

Dependent variables were:

- Total movement time (MT): time from when the object starts to move to when it is finally placed in the tray.
- Peak velocity.
- Peak velocity corrected by distance travelled (PVcorr): The peak velocity (PV) of a limb movement is generally related to the average speed of execution by the following association:

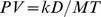
Where D is the distance moved, MT is the movement time and k is a scaling factor that reflects the shape of the velocity curve (e.g. for a sinusoid k = 1.57 whereas for an optimally smooth bell shaped curve k = ∼1.68.) If a participant completes a synchronous bimanual task by using the equivalent motor commands for both left and right hand movements, with equivalent MT envelopes, then the ratio of PV for the left and right movements should equate to the simple ratio of the distances travelled (i.e. 1.0 for equal distances). Where the distance to be travelled is not equal (20 cm vs. 60 cm movements to tray 1 or 3) then if the task is completed by re-scaling the same motor command structure the ratio of PVs for the left and right hands should still reflect the ratio of the distances travelled (3.0 or 0.33 e.g. one hand moves three times as fast as the other to travel 3 times as far). By computing the ratio of peak velocities for dominant vs. non-dominant limbs, corrected by the ratio of distance travelled for each limb 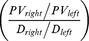 we get an estimate of how similar the central motor command structure was for both limbs (PVcorr).

- Deceleration time: time from when the object reaches peak velocity to the point at which it is finally placed in the tray.
- Hover time: time from when the hand decelerates to 10% of its peak velocity to when it finishes its approach to the tray (a subset of deceleration time). Riek et al [4] introduced a variable called ‘hover time’ as they noticed that movements appeared to exhibit an initial phase in which the limb was transported to the target, followed by a ‘hover’ phase in which the hand was held stationary above the target. They defined hover time as the time from the first minimum of the tangential speed to the end of the movement. As they were the first to introduce the hover time concept there is no accepted level. For this reason, along with the fact that much of this movement would probably be captured as part of what we are calling final adjustment time, hover time of the hand was established to start and end at specific velocity points. It was established as the time from which the hand reached a certain level of its peak velocity to the time the hand finished its approach to the object, and is therefore a sub-set of deceleration time. Two measures were taken to ensure an accurate reflection of the movement was established: 10% and 20% of the peak velocity were taken as the start of hover time. The finish of the approach of the hand was identified as the time point at which the hand reached 3% of its peak velocity. Results for both hover time measures were equivalent in terms of the main effects and interactions found, so only the 10% hover time measure is reported.
- Final adjustment time: time from finish of approach (3% of peak velocity) to final placement in the tray (another subset of deceleration time). Here we wanted to examine the final portion of the reach when the hand was at an extremely low velocity and likely to be waiting for the other hand to catch up. This has not been examined before so there is no accepted value.
- Number of adjustments made during the approach phase (peak velocity to finish of approach) and final adjustment phase (finish of approach to final placement). The adjustments were obtained using a custom written Matlab program to identify the zero crossings in acceleration.
- Synchrony of the hands at start, time to peak velocity, finish of approach and final placement: For each trial the time points at which the right hand reached these kinematic landmarks was subtracted from the left hand at the same landmark. Absolute values were used to form the means. This means that no account is taken of which hand started first, finished the approach or final placement first, or which hand reached peak velocity first. Absolute values were chosen because they are a better reflection of performance. If a participant was to finish a movement with his right hand 20 ms before his left on 5 trials, but finish with his left hand 20 ms before his right on the other 5 trials, he would obtain an exact synchrony mean of 0 ms between hands. The absolute mean would show 20 ms between hands. Clearly the exact value implies perfect synchrony and we know this was not the case. We also examined synchrony in line with the argument of Miller and Smyth [6]; that the analysis of mean data alone is insufficient to provide information about the nature of coordination, and data must be assessed by looking at the absolute differences between the limbs on a trial-by trial basis.

Mean values for each dependent measure were derived from the 10 experimental trials performed to each tray. The independent variables of interest are group (young, older), hand (right, left) and tray/distance (1, 2, 3). This produces a 2×2×3 design with each participant making reaches to all three trays. A series of mixed ANOVAs were used to test for statistical significance (α = 0.05). When a significant hand×tray interaction was found this was explored using paired samples t-tests to examine the right hand vs. the left hand at each tray. When a significant group×tray interaction was found this was explored using independent samples t-tests to examine the young group vs. the older group at each tray. If a significant 3-way interaction emerged for a dependent variable it was further examined by performing a separate 2 (hand)×2 (group) mixed ANOVA for the dependent variable to each of the three trays. For all dependent variables, when the sphericity assumption was violated F and p values generated using the Greenhouse-Geisser correction are reported.

## Results

**Figure 3:**
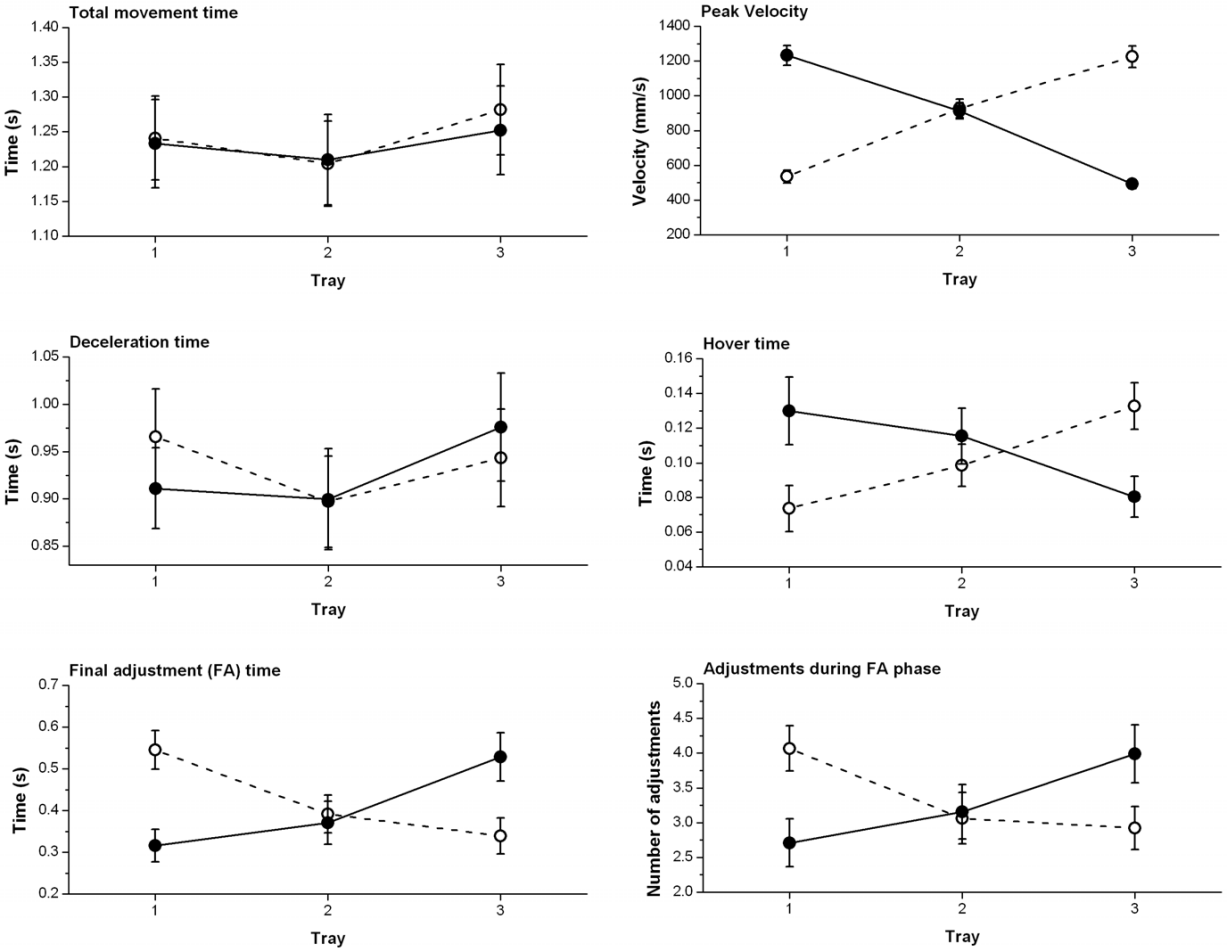
Significant hand×tray interactions. These plots show the dependent variables where significant hand×tray interactions were identified. For each plot the solid circles and solid line represent the right hand and the open circles and dotted line represent the left hand. Circles show the mean and error bars the standard error.

**Figure 4:**
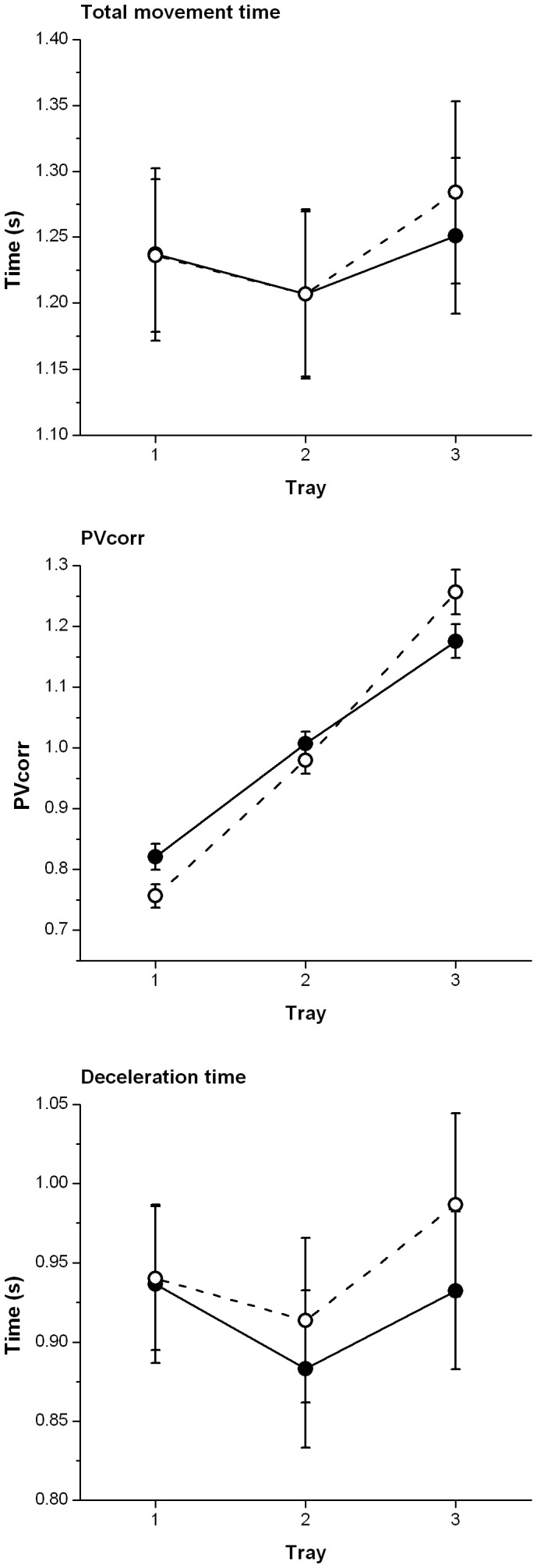
Significant group×tray interactions. These plots show the dependent variables where significant group×tray interactions were identified. For each plot the solid circles and solid line represent the young adults and the open circles and dotted line represent the older adults. Circles show the mean and error bars the standard error.

Means and standard deviations for all main effects (collapsed across the other independent variables) are reported in Table 1. The statistics for main effects and interactions are displayed in Table 2. Figure 3 shows the significant hand×tray interactions and Figure 4 shows the significant group×tray interactions.

**Table 1:** The means (and standard deviations) for all dependent variables.

Dependent variable	Young	Elderly	Left hand	Right hand	Tray 1	Tray 2	Tray 3
Total movement time (s)	1.239 (0.197)	1.242 (0.211)	1.242 (0.205)	1.239 (0.203)	1.247(0.191)	1.207(0.207)	1.267 (0.211)
Peak velocity (mm/s)	828 (321)	946 (336)	896 (330)	879 (339)	884 (385)	920 (163)	859 (402)
PVcorr	1.001 (0.165)	0.998 (0.240)			0.789 (0.070)	0.993 (0.070)	1.216 (0.135)
Deceleration time (s)	0.917 (0.164)	0.947(0.172)	0.935 (0.167)	0.929 (0.171)	0.938 (0.156)	0.898 (0.167)	0.960 (0.179)
Hover time (s)	0.125 (0.051)	0.085 (0.047)	0.101 (0.05)	0.108 (0.06)	0.102 (0.062)	0.106 (0.047)	0.107 (0.049)
Final adjustment (FA) time (s)	0.354 (0.170)	0.475 (0.167)	0.424 (0.171)	0.405 (0.187)	0.431 (0.182)	0.380 (0.159)	0.433 (0.192)
Adjustments during approach	1.330 (0.635)	0.885 (0.517)	1.002 (0.607)	1.214 (0.616)	0.980 (0.688)	1.220 (0.597)	1.123 (0.553)
Adjustments during FA phase	2.777 (1.082)	3.859 (1.257)	3.352 (1.217)	3.285 (1.364)	3.389 (1.297)	3.109 (1.250)	3.457 (1.320)
Synchrony at start (s)	0.038 (0.020)	0.045 (0.021)			0.042 (0.015)	0.032 (0.016)	0.051 (0.026)
Synchrony at peak velocity (s)	0.079 (0.039)	0.066 (0.027)			0.078 (0.035)	0.060 (0.028)	0.08 (0.037)
Synchrony at finish of approach (s)	0.213 (0.078)	0.166 (0.093)			0.240 (0.069)	0.117 (0.054)	0.211 (0.088)
Synchrony at final placement (s)	0.058 (0.021)	0.034 (0.024)			0.052 (0.029)	0.042 (0.022)	0.043 (0.025)

**Table 2:** The significant main effects and interactions; effect size is reported as partial eta squared.

Dependent variable	Main effect/interaction	d.f.	F-value	P-value	η_p_^2^
Total movement time	Tray	2,40	20.75	<0.001	0.509
	Group×Tray	2,40	4.18	<0.05	0.173
	Hand×Tray	2,40	7.06	<0.01	0.261
	Group×Hand×Tray	2,40	3.17	= 0.053*	0.137
Peak velocity	Group	1,20	3.91	= 0.062*	0.163
	Tray	2,40	23.36	<0.001	0.539
	Hand×Tray	1.11,22.26	1080.89	<0.001	0.982
PVcorr	Tray	2,40	308.41	<0.001	0.939
	Group×Tray	2,40	9.59	<0.001	0.324
Deceleration time	Tray	2,40	24.53	<0.001	0.551
	Group×Tray	2,40	4.06	<0.05	0.169
	Hand×Tray	1.26,25.29	16.44	<0.001	0.451
	Group×Hand×Tray	2,40	5.95	<0.01	0.229
Hover time	Group	1,20	12.06	<0.01	0.376
	Hand×Tray	2,40	13.79	<0.001	0.408
Final adjustment (FA) time	Group	1,20	4.40	<0.05	0.180
	Tray	2,40	11.43	<0.001	0.364
	Hand×Tray	2,40	109.46	<0.001	0.846
Adjustments during approach	Group	1,20	6.88	<0.05	0.256
	Hand	1,20	5.80	<0.05	0.225
	Tray	1.41,28.26	4.17	<0.05	0.172
Adjustments during FA phase	Group	1,20	6.82	<0.05	0.254
	Tray	2,40	5.13	<0.01	0.204
	Hand×Tray	2,40	68.70	<0.001	0.775
Synchrony at start	Tray	2,40	6.57	<0.01	0.247
Synchrony at peak velocity	Tray	2,40	4.20	<0.05	0.174
Synchrony at finish of approach	Group	1,20	5.41	<0.05	0.213
	Tray	2,40	24.09	<0.001	0.546
Synchrony at final placement	Group	1,20	14.21	<0.01	0.416

* *Denotes a result approaching significance.*

### Total Movement Time

There was no significant main effect of group or hand but there was a significant main effect of tray. Bonferroni pairwise comparisons showed this main effect was driven by differences between the overall MT to trays 1 and 2 (p<0.01) and trays 2 and 3 (p<0.01) but not between trays 1 and 3, and MT was longest to tray 3 and shortest to tray 2. There was a significant hand×tray interaction along with a significant interaction between group and tray and a marginally significant group×hand×tray interaction. For this reason we examined the group and hand at each tray separately. For tray 1 no main effect of group or hand was identified, but a marginally significant group by hand interaction emerged [F(1,20) = 4.687; p = 0.043, η_p_^2^ = 0.19] but did not withstand alpha level adjustment. For tray 2 no main effects or interactions were identified. For tray 3 only a main effect of hand emerged [F(1,20) = 9.037; p<0.01, η_p_^2^ = 0.31], with the total MT of the left hand being greater than that of the right.

### Peak Velocity

Older adults reached higher peak velocities than the young, but this trend did not reach levels of conventional significance (p = 0.062). No significant main effect of hand was identified. A significant main effect of tray was established with Bonferroni pairwise comparisons showing significant differences between the peak velocities to all trays (p<0.05); highest to tray two and lowest to tray three. A hand×tray interaction was identified and subsequent paired samples t-tests showed the peak velocity of the right hand was significantly greater than that of the left in reaches to tray 1 [t(21) = −42.91; p<0.001] and smaller than that of the left in reaches to tray 3 [t(21) = 25.406; p<0.001]. There was no significant difference in peak velocity of the hands in movements to tray 2. No further significant interactions emerged.

### PVcorr

Reaches to tray 2 were characterised by PVcorr values of around 1, showing the peak velocities of the left and right hands to be very similar. In reaches to tray 1 the lower PVcorr values (<1.0) represent reaches where the right hand is moving faster than the left, but not three times as fast. In reaches to tray 3 the higher PVcorr values (>1.0) represent reaches where the left hand is moving faster than the right, but not three times as fast. The ANOVA revealed a significant main effect of tray with differences between PVCorr values to all trays (highest to tray 3 and lowest to tray 1, p<0.05). There was no main effect of group but there was a significant group×tray interaction. Independent samples t-tests showed the groups differed significantly in reaches to the first [t(20) = 2.26; p<0.05] tray only, with young adults producing PVcorr scores closer to 1 then the older adults. The young group were more capable of scaling up/down the velocity of their hands to compensate for the distance travelled, whereas the older adults did not do so quite as effectively.

### Deceleration Time

No significant main effects of group or hand emerged. There was a significant main effect of tray, with deceleration time being longest to tray 3 and shortest to tray 2. Bonferroni pairwise comparisons revealed significant differences between the deceleration times to trays 1 and 2 (p<0.01), and trays 2 and 3 (p<0.001) but not between trays 1 and 3. There was a significant hand×tray interaction, a significant group×tray interaction, and a significant group×hand×tray interaction. For this reason we examined the group and hand at each tray separately. For tray 1 no main effect of group was identified, but a significant main effect of hand [F(1,20) = 20.78; p<0.001, η_p_^2^ = 0.51] emerged, with the deceleration time of the left hand being greater than that of the right, along with a significant group×tray interaction [F(1,20) = 5.281; p = 0.032, η_p_^2^ = 0.21] although this did not withstand alpha level adjustment. For tray 2 no main effects or interactions were identified. For tray 3 only a main effect of hand emerged [F(1,20) = 7.050; p<0.05, η_p_^2^ = 0.261], with the deceleration time of the right hand being greater than that of the left.

### Hover Time

A significant main effect of group was established with the young hovering for longer than the older adults. A significant hand×tray interaction was identified and paired samples t tests showed the hover time of the right hand was significantly longer than that of the left in reaches to tray 1 [t(21) = −3.604; p<0.01] and shorter than that of the left in reaches to tray 3 [t(21) = 5.266; p<0.001]. There was no significant difference between the hands in reaches to tray 2. No other significant interactions were established.

### Final Adjustment (FA) Time

A significant main effect of group was found, with the older adults spending longer in the FA phase than the young. There was also a significant main effect of tray and Bonferroni pairwise comparisons revealed significant differences between the FA times to trays 1 and 2 (p<0.001) and trays 2 and 3 (p<0.01) but not trays 1 and 3, with FA time being longest to tray 3 and shortest to tray 2. A significant hand×tray interaction was identified and paired samples t-tests revealed the FA time of the right hand was significantly shorter than that of the left in reaches to tray 1 [t(21) = 10.348; p<0.001] and longer than that of the left in reaches to tray 3 [t(21) = −8.767; p<0.001]. There was no significant difference between the hands in reaches to tray 2, and no other significant interactions.

### Adjustments during Approach Phase

Heterogeneity of variance demanded the data were transformed using a natural log transformation in order to carry out statistical analyses. A significant main effect of group was found, with the young making more adjustments than the older adults. There was also a significant main effect of hand, with the right making more adjustments than the left, and tray, with the number of adjustments being greatest to tray 2 and smallest to tray 1. Bonferroni pairwise comparisons revealed significant differences between trays 1 and 2 (p<0.01) but not between trays 1 and 3 or 2 and 3. No significant interactions emerged.

### Adjustments during Final Adjustment (FA) Phase

A significant main effect of group was found with the older adults making more adjustments than the young. No main effect of hand emerged but there was a significant main effect of tray, and Bonferroni pairwise comparisons showed that this was driven by significant differences between the number of adjustments to trays two and three only (p<0.05). The number of adjustments was greatest in reaches to tray 3 and smallest in reaches to tray 2. A significant interaction of hand and tray was found and paired samples t-tests showed the number of adjustments made by the right hand to be significantly smaller than the number made by the left hand in reaches to tray 1 [t(21) = 9.010; p<0.001] but significantly greater than the number made by the left hand in reaches to tray 3 [t(21) = −6.261; p<0.001]. There was no significant difference between the hands in reaches to tray 2.

### Synchrony

For synchrony in starting to move there was no main effect of group but there was a main effect of tray, and Bonferroni pairwise comparisons showed this was driven by significant differences between the reaches to trays 1 and 2 (p<0.05) and trays 2 and 3 (p<0.01) but not between trays 1 and 3. Reaches were most synchronous to tray 2 and least to tray 3. No significant interaction of group and tray emerged. The same pattern emerged for synchrony in time to peak velocity: there was no significant main effect of group but a significant main effect of tray was found and Bonferroni pairwise comparisons showed this was driven by significant differences between the reaches to trays 1 and 2 (p<0.05) and trays 2 and 3 (p<0.05) but not between trays 1 and 3. Again, reaches were most synchronous to tray 2 and least to tray 3. No significant interaction of group and tray emerged. For synchrony of the hands at the finish of approach a significant main effect of group emerged with the young being more asynchronous than the older adults. A significant main effect of tray was identified and Bonferroni pairwise comparisons showed there were significant differences between tray 1 and 2 (p<0.001) and between 2 and 3 (p<0.001) but not between trays 1 and 3. Reaches were most synchronous to tray 2 and least to tray 1. There was no significant interaction between group and tray. For synchrony of the hands in their object placement a significant main effect of group was found with the young being more asynchronous than the older adults. No significant main effect of tray was identified and there was no significant interaction between group and tray.

## Discussion

The aims of this experiment were to discover whether the older adults produced bimanual reaches that differed from those of the young, and also whether the two groups were differentially affected by asymmetrical compared to symmetrical bimanual movements. Unlike in previous experiments, here precision was key and participants specifically instructed to place the objects simultaneously.

In terms of group effects, the results of the overall movement time of reaching movements show that the two groups did not significantly differ. This is in direct contrast to the findings of Stelmach et al [12] (and our predictions) who found that the older adults took significantly longer to complete their reaching movements in both unimanual and bimanual conditions than the young. Key differences between this experiment and that of Stelmach et al [12] might be behind these contrasting findings. These include type of movement (pointing v carrying), direction of movement (sagittal v lateral), accuracy demands, and instruction. Accuracy was not assessed in Stelmach et al’s experiment, and one assumes pointing could be fairly imprecise. In this study participants had to be accurate in order to fit both objects in the tray, and these accuracy demands may have prompted the young to slow down and produce a movement more similar to the older adults, perhaps because they could not rely on relatively efficient feedforward strategies as they might usually be able to were accuracy requirements reduced.

Although the overall movement times did not differ between groups, the reach kinematics of the groups were parameterised differently. The older adults reached higher peak velocities than the young (although this difference just failed to reach levels of conventional significance), spent less time in the hover phase of the reaches, and made fewer adjustments during the approach phase. In contrast, the older adults spent longer than the young in the final adjustment phase of the reach and made more adjustments at this point, so the pattern of outcomes for final adjustment times was the opposite of that obtained for hover times. The young spent longer in the hover phase, made more corrections during this phase, and hence arrived at the tray with a greater degree of accuracy. They may have been able to utilise online feedback during the reach, and correct trajectory errors as they moved, although further evidence would be required to support this theory. In contrast, the older adults moved the object to the tray’s vicinity as fast as possible and without much accuracy, with the result that they spent longer right at the end of the reach (in the final adjustment phase), when they could visually monitor both objects, correct trajectory errors and place the objects in the tray. These results highlight the importance of examining the final stages of the reach separately. Group differences can get lost if the entire deceleration phase is considered as a whole.

These data imply that older adults might initially use feedforward strategies for the completion of bimanual tasks such as this. In a previous unimanual study [9] we found that older adults seemed to rely to a greater degree on closed-loop visual control as they were detrimentally affected to a greater extent than their younger counterparts when vision of the hand was removed during the reach. In the current experiment both hands could not be foveated until they had been moved close together, and for this reason the older adults may have had to rely on other means of feedback (such as proprioception), or feedforward strategies, until they could visually monitor both objects and correct trajectory errors.

As well as age-related differences in the use of visual control, previous research has highlighted that proprioceptive abilities change with age. Older adults show a significant decline in position sense with age, are less capable of sensing joint motion, and are more variable in terms of their ability to monitor joint position during motion (see [14] for a comprehensive review). These declines in proprioceptive abilities may help explain some of the group differences that emerged in this study. Without efficient proprioceptive control one becomes more reliant on feedback from other areas, most notably vision. In the bimanual task featured here vision of the objects is not concurrently available until they are close to the target tray. Up until this point reaches are presumably guided by feedforward strategies, or proprioception. If proprioceptive abilities are reduced (as they are in older adults) then vision becomes more important, and if you cannot foveate the targets until late on in the movement you might have to wait until this point to correct trajectory errors. This is in line with our findings that the older adults spent a significantly longer time in the final phase of the reach than their younger counterparts, and also made more adjustments during this phase, perhaps compensating for trajectory errors caused by less efficient proprioceptive abilities and/or use of feedforward control.

The older adults were actually more synchronous than the young in terms of placing the objects in the trays and also in finishing their approaches to the trays. There were no significant differences between the groups in terms of the synchrony of the hands in the time they took to start the movements or reach peak velocity. These findings are in contrast with those of Stelmach et al [12] who found that the older adults were less synchronous than the young in starting and terminating the two hands in bimanual movements. Again this could be due to different task demands, as with the overall movement time findings, although further analysis is needed to support this speculation. It is likely that instruction played a vital role here. In contrast with traditional methods, we asked participants to try and place the objects in the tray at the same time. We felt it important to investigate whether older adults could place objects synchronously when specifically asked to do so, and it seems that they can. The fact that they were actually more synchronous than the young at finish of approach and placement might merely reflect the fact that they took the instruction more seriously.

The lack of group by tray interactions found for most of the dependent variables examined shows that the older adults were no more detrimentally affected than the young by the asymmetric bimanual movements compared to the symmetric ones. Even for movement time and deceleration time, where group by tray interactions did emerge, no simple main effects were found. These findings are in agreement with those of Stelmach et al [12] who found that the older adults showed equivalent changes to the young when asymmetry was introduced. In contrast, others found that older adults showed specific difficulties (often in terms of greater variability) in motor tasks when the two hands were moving independently/asynchronously [15], [16], [17]. Bangert et al [15] postulated that the more simple synchronous tasks could be performed relatively automatically, whereas the more difficult asynchronous condition caused a greater reliance on attentional resources and executive control. Fling et al [16] also supported this theory as group differences were restricted to the more difficult movements requiring higher levels of interhemispheric inhibition (IHI). Our contrasting findings might be due to the fact that the tasks employed in the other studies (discrete tapping [15], [16], and force production [17]), were quite different to those utilised in the current experiment.

A group by tray interaction was identified for PVcorr, a measure of the ratio of peak velocity between the two hands corrected by distance travelled that provides an estimate of how similar the central motor command structure was for both limbs. Further analyses revealed group differences in reaches to tray 1. Neither group moved the hand that had to travel further quite fast enough, but this was exaggerated in the older adults, which might reflect a less efficient motor plan, or a misestimate of distance. We believe that the introduction of this new variable is a useful contribution to the field of prehension research and is one that should be examined in future studies along with the more traditional kinematic variables.

Recent research has shed some light on the underlying neural mechanisms behind age differences in bimanual prehension tasks. As mentioned in the Introduction, Heuninckx et al [11] discovered that in a test of interlimb coordination in older adults performance was correlated with activation not only in the classic motor coordination regions but also frontal and higher level sensorimotor regions, reflecting greater cognitive control and greater reliance on sensory information processing respectively. The authors suggested this additional recruitment led to an increase in performance for some subjects, and is consistent with the idea that additional activation compensates for age-related decline in brain function. Age differences in activation levels can also be seen in the work of Fling and colleagues. When tasks were difficult (asynchronous and therefore with increased interhemispheric inhibition requirements) larger corpus callosum (CC) size and better CC microstructure of relevant subregions was correlated with poorer performance in young adults but better performance in older adults [16]. These findings indicate that age differences in these callosal microstructure fibres are important contributors to bimanual control [16], [17].

In terms of how synchronous the hands were, results are mixed with regards to an effect of distance moved. Synchrony of final placement was unaffected by distance. This is in direct contrast to the findings of Stelmach et al [12] who found that both age groups in his study were similarly more asynchronous in terminating asymmetric compared to symmetric movements, and Bingham et al [1] who found the asynchronies present on arriving at the target remained to the end of the reach-to-grasp movement. Again, this is likely to be because we stressed the importance of placing the objects down synchronously. For synchrony at start, time to peak velocity, and finish of approach to the tray the movements were significantly more synchronous in reaches to tray 2 than they were to trays 1 and 3. In this case the symmetrical movement required to reach tray 2 allowed the participants to move their hands in a more synchronous manner. These findings are in line with the idea that participants move more synchronously when both hands are moving the same distance, and are therefore also in line with the findings of Stelmach et al [12] and Bingham et al [1].

Bingham et al [1] found some evidence for asynchronies in arriving at the object caused by reaching for targets at different distances, and interestingly even when reaching for targets at the same distance participants produced reaches that were asynchronous in arrival times. Similarly, in this experiment neither group is perfectly synchronous even when reaching to the middle tray when both hands moved the same distance. Bingham et al [1] suggested that such asynchronies might be a result of a need for visual information to guide each hand in turn. The importance of vision in guiding bimanual reaches has been recently highlighted by Srinivasan and Martin [18] who examined the relationship between visual feedback and synchrony of the two hands in symmetric bimanual reach movements. The authors found that although reaches were synchronous up until the point of peak velocity, the degree of synchrony in the terminal phases of the movements was significantly reduced, probably because of a reliance on visual feedback and the fact that the two hands could not be fixated simultaneously. This was evidenced by their identification of four distinct eye-hand coordination patterns (terminal, selective, predictive and intermittent gaze strategies; see paper for a full review) that emerged as a result of task demands. It would be interesting to examine the eye movement strategies of older adults in a task similar to that of Srinivasan and Martin [18]. It may be that they show markedly different strategies to younger adults due to an overreliance on visual feedback: perhaps, for example, never producing the predictive gaze strategy (where gaze is shifted to the second target before the first is placed).

### Conclusions

When older and younger adults were instructed to perform a bimanual movement with simultaneous placement it was discovered that the groups did not differ in terms of overall movement time or deceleration time, and the older adults were able to complete the asymmetric bimanual movements as effectively as their younger counterparts. Nevertheless, it does seem that the older adults produced reaches that were designed to reach the vicinity of the tray quickly, after which they made discreet adjustments to their trajectories in order to place the objects in the tray correctly. The young moved more slowly to utilise online feedback and make adjustments during the approach, so were more accurate when they finally reached the tray, as evidenced by the fact that fewer adjustments were then required. These findings are consistent with the idea that older adults have problems using online control (be it visual [10], or proprioceptive [14]), but rather than manifesting themselves in the large section of reach following peak velocity, these deficits are not apparent until the final section of a reach. Older adults can obviously complete some fine motor control tasks in a similar time to their younger counterparts, but they do so in a qualitatively different way, perhaps to overcome deficits with online control that come with aging.
